# Age- and Varicose Disease-Associated Changes in the Muscular Components of the Great Saphenous Vein

**DOI:** 10.3390/jcm14186448

**Published:** 2025-09-12

**Authors:** Khurshed A. Abduvosidov, Irina A. Chekmareva, Valeria G. Shestakova, Irina N. Shabanova, Alexander G. Alekseev, Edgar S. Kafarov, Alexander A. Palalov, Irina N. Yashina, Evgeny N. Galeysya

**Affiliations:** 1 Department of Human Morphology, Russian Biotechnological University, Volokolamsk Highway, 11, Moscow 125080, Russia; sogdiana99@gmail.com (K.A.A.);; 2 Department of Anatomy, Histology and Embryology, Tver State Medical University, Sovetskaya Str., 4, Tver 170100, Russiair.nik@gmail.com (I.N.S.); 3 Diagnostics’ Department, Moscow Clinical Scientific and Practical Center Named After A.S. Loginov, Highway Enthusiasts, 86, Moscow 111123, Russia; 4 Electron Microscopy Laboratory, A. V. Vishnevsky National Medical Research Center of Surgery, Bolshaya Serpukhovskaya Str., 27, Moscow 117997, Russia; 5 Department of Human Anatomy, Peoples’ Friendship University of Russia, Miklukho-Maklaya Str., 6, Moscow 117198, Russia; galeysya-en@rudn.ru; 6 Department of Normal and Topographic Anatomy with Operative Surgery, Kadyrov Chechen State University, A. Sheripova Str., 32, Grozny 364907, Russia; edgar.kafaroff@yandex.ru; 7 Department of Human Anatomy, Kursk State Medical University, Karla Marksa Str., 3, Kursk 305041, Russia

**Keywords:** great saphenous vein, varicose, aging, chronic venous disease, vascular remodeling

## Abstract

Varicose disease and other age-related vascular illnesses are extremely prevalent among the adult population. Despite this, research devoted to involutive changes in the veins of the lower extremities is rare and fragmented. Complex morphological evaluation of the wall of the vein related to age and varicose disease can add valuable data to fundamental geriatric and vascular medicine. **Objectives:** The study was designed to determine the age-related changes in the muscular component of the great saphenous vein and changes associated with varicose disease. **Materials and Methods:** A morphological study of a specimen of the great saphenous vein was conducted on 55 deceased individuals and 80 patients with varicose disease. Four age subgroups were identified: young, middle-aged, elderly, and senile. A total of 135 fragments of the great saphenous vein were evaluated. Histological, morphometric, and electron microscopic studies were performed. A quantitative analysis of the volumetric fraction of muscular components was calculated using the Shapiro–Wilk test, Kruskal–Wallis (ANOVA) and Mann–Whitney methods with Bonferroni correction. **Results:** Our study showed that the amount of connective tissue elements between bundles of smooth muscle cells increased with age. In patients with varicose disease, we observed an appearance of connective tissue fibers among smooth muscle cells, more pronounced with the disease progression. The structure of smooth muscle cell changes. Thus, we observed hypertrophy and phenotypic heterogeneity of cells with subsequent destruction of communicative contacts. The values of subintimal longitudinally arranged smooth muscle cells reached their maximum in middle age in both normal and varicose veins, while significant decrease occurred in elderly and senile patients. Quantitative indicators of circularly arranged smooth muscle cells of the middle layer did not change with age but significantly decreased in varicose disease. Age-related changes are characterized by an increase in the proportion of smooth muscle cells in the outer layer. In varicose veins, in young and middle-aged patients, the content of bundles of longitudinally arranged smooth muscle cells in the outer layer was higher compared to the age norm, with a significant decrease in senile age. **Conclusions**: The age norm of the muscular component of the great saphenous vein wall is characterized by an increase in the volumetric fraction of subintimal longitudinally arranged smooth muscle cells in middle age, the volumetric fraction of circularly arranged smooth muscle cells of the middle layer remains unchanged, and the volumetric fraction of bundles of longitudinally arranged myocytes of the outer layer increases. With age in varicose disease, sclerotic changes progress in the structure of the great saphenous vein at the tissue, cellular, and intracellular levels, leading to a decrease in the volumetric fraction of all muscular components of the great saphenous vein structure.

## 1. Introduction

Despite significant advancements in medical diagnostics, prevention, and treatment, varicose disease (VD) of the lower extremities remains an extremely prevalent peripheral vascular disorder, frequently progressing to chronic venous insufficiency (CVI) and trophic complications [1,2]. The elderly and senile populations are particularly affected, with a high incidence of CVI and a substantial risk of developing trophic ulcers (exceeding 5% in some cases) [3]. Notably, healthcare expenditure for patients over 60 years old is seven times higher than for younger individuals, underscoring the economic burden of this condition.

The pathogenesis of VD remains a subject of ongoing research and debate [4]. While some studies attribute the disease to venous valve dysfunction, resulting in pathological reflux, others emphasize structural alterations in the venous wall—including smooth muscle cell (SMC) reorganization and collagen-elastin imbalances—leading to wall weakening and venous dilation [5]. These changes trigger an inflammatory cascade, beginning in the endothelium and eventually involving the entire venous wall [6]. Hemodynamic overload further exacerbates venous valve dysfunction, promoting reflux through the sapheno-femoral, sapheno-popliteal, or perforating veins. Consequently, excessive blood pooling in the superficial venous system drives progressive wall remodeling and varicosity formation [4,7]. The wall of the vein remains in an extremely stretched state, resulting in the structural remodeling of the vessel. The scientific literature addresses the morphology of the vein in varicose disease [8,9]; however, research focused on the involutive changes in the veins of the lower extremities in old-aged patients and elderly are rare (60 to 74 and 75 to 89 years, respectively).

## 2. Materials and Methods

A morphological study was conducted using autopsy and biopsy material from fragments of the great saphenous vein (GSV) obtained at the level of the upper third of the thigh (Table 1). Autopsy material was collected from 55 individuals (29 females and 26 males) without pathological changes in the lower extremity venous system at the time of death. A review of the deceased patients’ medical records revealed no documented history of venous disorders. Gross examination of the lower limbs during autopsy also showed no visual evidence of varicose veins or clinical signs indicative of chronic venous disease or insufficiency. Biopsy material was obtained during phlebectomy from 80 patients (44 females and 36 males) diagnosed with varicose disease (clinical classes 4–6 of chronic venous disease according to the CEAP classification). The criterion for obtaining a biopsy of the great saphenous vein was the presence of dilation and valvular incompetence in the trunk of the great saphenous vein near the sapheno-femoral junction, as confirmed by preoperative Doppler ultrasound angioscanning. During the surgical procedure, following the exposure of the vein trunk and the sapheno-femoral junction, a crossectomy was performed (ligation and transection of the great saphenous vein trunk and its tributaries at the junction). Subsequently, a fragment of the great saphenous vein was excised for biopsy, followed by a stripping procedure.

All patients provided written informed consent for medical treatment. The use of the material obtained in the course of said medical treatment did not require additional consent. The autopsy material was sourced from deceased patients at the Moscow Clinical Scientific and Practical Center named after A.S. Loginov. The study utilized samples and data from patients who had provided written informed consent for both treatment and the future use of their medical information in research, as approved by the Local Ethics Committee (protocol No. 6/2021, dated 23 June 2021).

The samples were divided into four subgroups based on the age of deceased individuals and operated patients, following the WHO classification. Histological examination of GSV biopsies was performed using standard protocol, specimen were stained with hematoxylin-eosin using the Van Gieson method. Quantitative analysis assessed the volumetric fraction (VF) of muscular components, including longitudinally arranged (subintimal) smooth muscle cells (SMCs), circularly arranged SMCs of the middle layer and longitudinally arranged SMC bundles of the outer layer. VF was determined using the morphometric technique by G.G. Avtandilov. For each structure, ten random overlays of an ocular grid (comprising 4 squares with 100 test points each, totaling 1000 points) were evaluated. VF was calculated as the percentage of test points coinciding with the studied structures relative to the total points analyzed.

Additionally, electron microscopic examination was performed on 133 GSV fragments from 19 varicose disease patients. Then, specimens were fixed in 2.5% glutaraldehyde, post-fixed in 1% osmium tetroxide, dehydrated, and embedded in araldite resin. Semi-thin sections were prepared using an LKB V ultramicrotome (Sweden), followed by ultra-thin sections that were contrasted and examined under a JEM 100 CX electron microscope (JEOL, Tokyo, Japan) at 80 kV.

Preliminary statistical analysis (Shapiro–Wilk test, *p* > 0.05) indicated non-normal distribution of data; thus, VF results are presented as median and interquartile ranges (25%, 75%). Intergroup comparisons employed non-parametric methods: the Kruskal–Wallis test (ANOVA) for initial screening (*p* < 0.05 considered significant), followed by pairwise Mann–Whitney tests with Bonferroni correction (k = 0.05/6 = 0.0083; significance threshold *p* < 0.005).

## 3. Results

**Figure 1:**
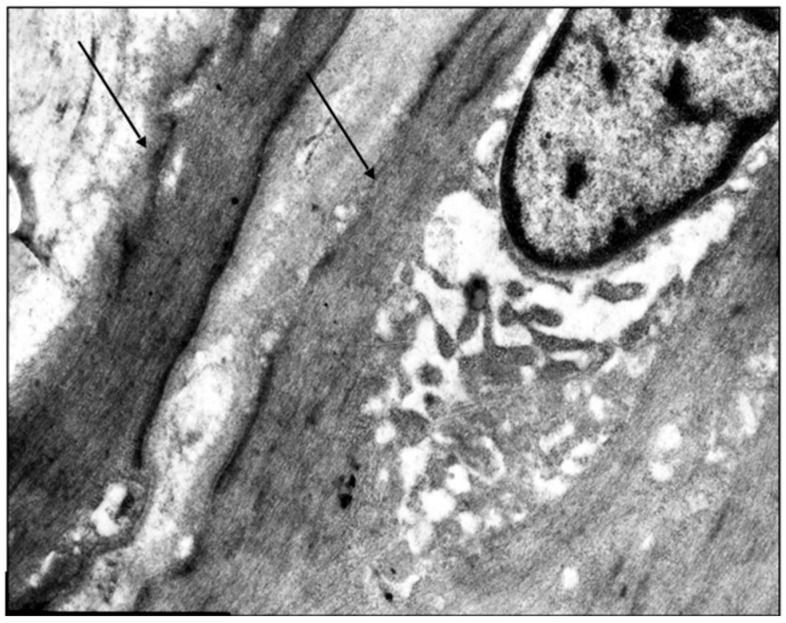
Fragment of the great saphenous vein in a young patient. Transmission electron microscopy. Magnification × 14,000. Hypertrophy of circularly arranged smooth muscle cells (arrows).

The venous wall’s primary muscular component consists of the middle layer’s circular smooth muscle cells, which are tightly packed in younger individuals. The subintimal longitudinally oriented smooth muscle cells within this layer show considerable variation in their presence and degree of development. In young patients with early stage varicose disease, typical smooth muscle cells are absent at the junction between the circular and longitudinal layers of the great saphenous vein’s middle layer. Instead, only twisted spiral nuclei are observed. Connective tissue fibers form thin strips between the circular smooth muscle cells, which themselves exhibit hypertrophic changes (Figure 1).

**Figure 2:**
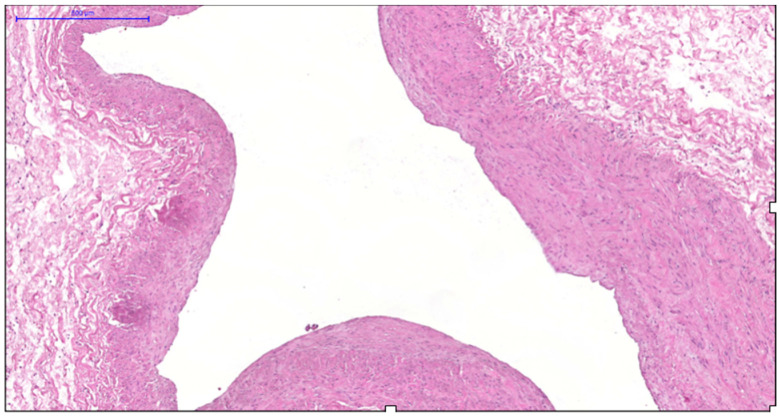
Fragment of the great saphenous vein in individuals of the second age group. Hematoxylin and eosin staining. Magnification × 100. Segment of the vein with minimal and maximal wall thickness.

**Figure 3:**
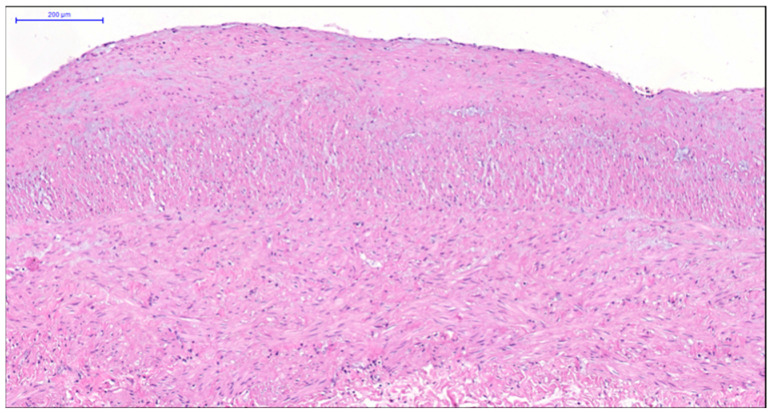
Fragment of the great saphenous vein in individuals of the second age group. Hematoxylin and eosin staining. Magnification × 200. Clearly defined layer of longitudinally oriented smooth muscle cells of the middle layer.

More pronounced structural alterations appear in the great saphenous vein of middle-aged and elderly individuals under normal conditions. Age-related changes manifest as uneven wall thickness and structural heterogeneity. Areas with thinner walls show reduced proportions of longitudinal and circular smooth muscle layers, while thicker regions contain these layers at maximal density (Figure 2 and Figure 3). The circular smooth muscle bundles become increasingly separated by connective tissue, creating a disorganized appearance.

**Figure 4:**
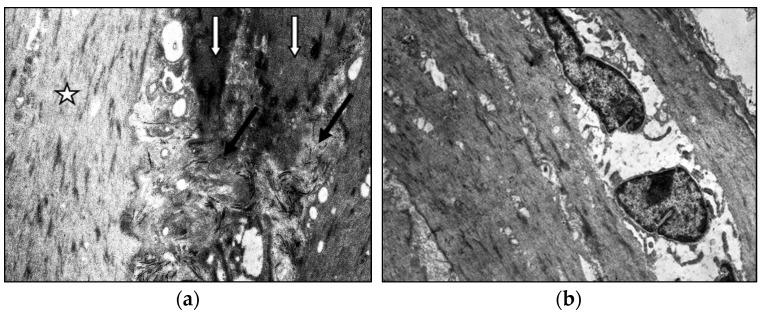
Fragment of the great saphenous vein in the second age group with a disease duration of more than 10 years. Transmission electron microscopy. (**a**)—collagen fibrils (black arrow) between “dark” smooth muscle cells (white arrow) and unchanged smooth muscle cells (asterisk) magnification × 12,000; (**b**)—“light” smooth muscle cells. Magnification × 14,000.

Middle-aged patients with VD demonstrate myoelastofibrosis and diffuse sclerosis within the venous wall, marked by expanded connective tissue layers between muscle bundles. Ultrastructural analysis reveals disorganized collagen fibrils and elastic fibers interspersed among phenotypically heterogeneous smooth muscle cells. In patients with decade-long varicose disease, electron microscopy identifies distinct “light” and “dark” myocytes (Figure 4). The “light” variants show reduced contractility due to organelle disarray and myolamellar breakdown, while “dark” cells display compressed, electron-dense cytoplasm with deformed myofilaments, reflecting cellular dysfunction and impaired intercellular communication.

**Figure 5:**
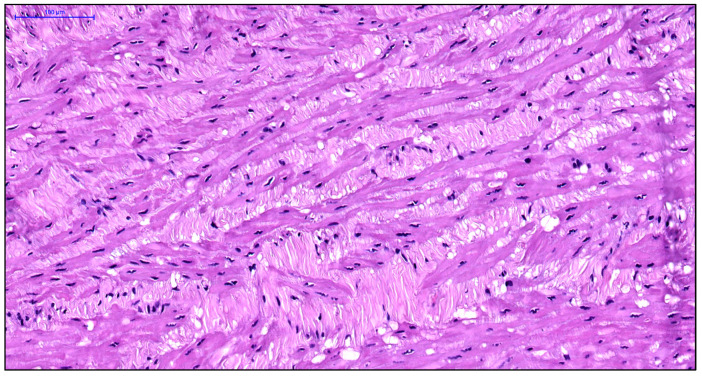
Fragment of the great saphenous vein in a patient from the third age group. Hematoxylin and eosin staining. Magnification × 400. Segment of the middle layer, notable disruption of structure and orientation of smooth muscle cell bundles. Fragmentation of smooth muscle cell fibers due to the growth of connective tissue between them in the form of coarse fibrous structures.

**Figure 6:**
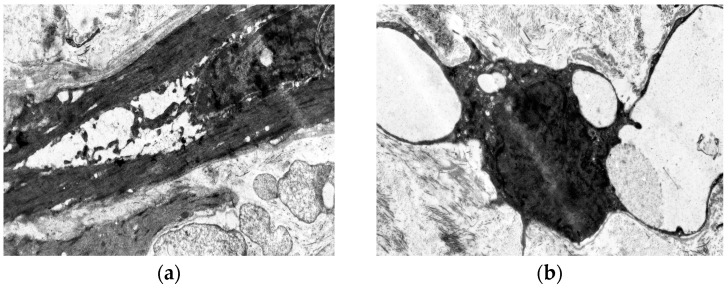
Fragment of the great saphenous vein in a patient from the third age group with a disease duration of more than 10 years. Transmission electron microscopy. (**a**)—“dark” smooth muscle cell surrounded by collagen fibers. Magnification × 9000; (**b**)—vacuolization of the cytoplasm of the “dark” smooth muscle cell. Magnification × 9000.

Elderly patients with VD exhibit disrupted myocyte orientation in the subintimal longitudinal layer. Connective tissue proliferation fragments muscle bundles, while the circular layer shows advanced diffuse sclerosis with scar formation (Figure 5). Ultrastructurally, degenerated “dark” myocytes dominate, surrounded by connective tissue infiltration (Figure 6).

**Figure 7:**
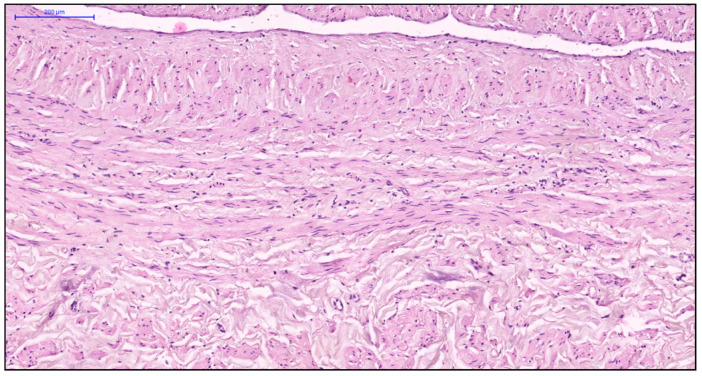
Fragment of the great saphenous vein in individuals of the fourth age group. Hematoxylin and eosin staining. Magnification × 100. Disorganization of the longitudinal and circular layers of smooth muscle cells of the middle layer.

Age-related involution produces alternating zones of tightly packed and sparsely distributed smooth muscle cells within the circular layer, highlighting its structural inconsistency (Figure 7).

**Figure 8:**
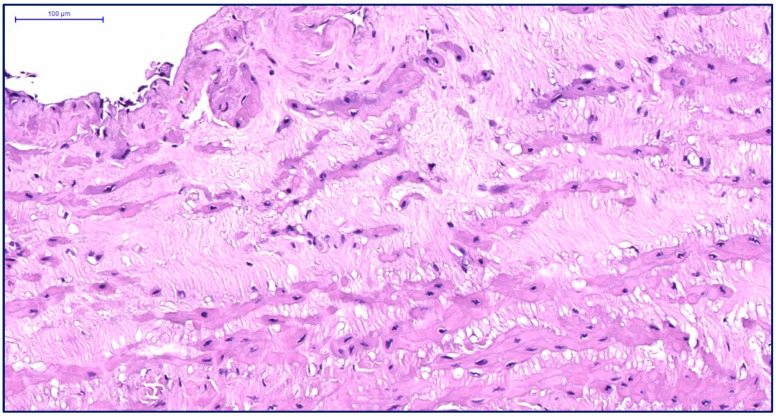
Fragment of the great saphenous vein in a patient from the fourth age group. Hematoxylin and eosin staining. Magnification × 200. The thickness of the inner layer is minimal, while the middle layer is thickened.

**Figure 9:**
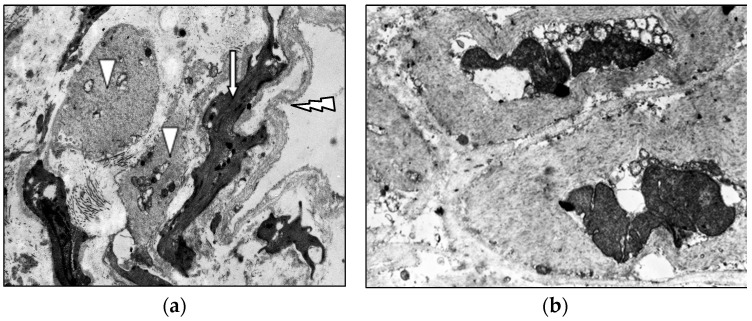
Fragment of the great saphenous vein in a patient from the fourth age group with a disease duration of five years. Transmission electron microscopy. (**a**)—Basal membrane delamination (arrow); “dark” smooth muscle cells (white arrow); “light” smooth muscle cells (triangle). Magnification × 9000; (**b**)—“light” smooth muscle cells; focal lysis of the matrix. Magnification × 9000.

Elderly varicose veins contain extensive fibrous regions housing atrophic, randomly oriented muscle bundles, indicative of sclerotic progression (Figure 8). Most myocytes are “dark” cells with elongated cytoplasmic processes; residual “light” cells contain pale mitochondrial matrices and focal cytoplasmic lysis (Figure 9), confirming progressive degenerative changes.

Quantitative analysis reveals age-dependent shifts in the great saphenous vein’s muscular composition. Under normal conditions, middle-aged individuals show significantly higher volumetric fractions of subintimal longitudinal smooth muscle cells than other age groups (Table 2 and Table 3). Varicose disease produces similar peak values in middle age, but elderly patients demonstrate marked reductions. While young and middle-aged varicose patients exhibit increased longitudinal muscle fractions compared to age-matched controls, elderly patients show declines coinciding with involutional changes.

## 4. Discussion

As noted by Vankov V.N., smooth muscle cells serve as crucial structural components of blood vessels, maintaining active tone and regulating vascular lumen diameter [10]. These muscular elements provide essential resistance to venous wall stress, preventing pathological vasodilatation. The circular muscle layer facilitates lumen constriction, while the longitudinal layer counteracts both this constrictive force and external vascular pressure. Several researchers have observed that normal aging involves involutional remodeling across all layers of the great saphenous vein, including its middle muscular layer [10,11,12].

The pathological changes associated with varicose disease differ markedly from normal age-related remodeling. As demonstrated by Badier-Commander C et al. [12] and other investigators, varicose transformation produces distinct structural alterations characterized by fibrosis and sclerosis [8,13]. The venous wall develops heterogeneous regions alternating between hypertrophy and atrophy. Hypertrophied zones exhibit transformed smooth muscle cells with diminished contractile capacity and enhanced proliferative activity, while atrophic areas show proportional reductions in both muscular and matrix components. Multiple studies confirm that varicose disease promotes connective tissue matrix degradation, marked by increasing collagen content and decreasing elastin within the venous wall [14,15,16].

Current research by Luis Cristovao Porto and others indicates that middle-layer remodeling in varicose disease progresses according to both patient age and disease duration. Comparative analyses reveal significant reductions in elastic fiber density and smooth muscle cell concentration compared to normal veins, supporting the venous wall insufficiency hypothesis of varicose pathogenesis [16]. Our investigation of the great saphenous vein’s muscular components has delineated both normative age-related changes and pathological alterations in varicose disease.

The impairment of elastic and contractile properties of the venous wall and the reduction in its tone is likely associated with the appearance of “dark” and “light” smooth muscle cells (SMCs), which reflect different types of cellular damage. SMC hypertrophy, as a universal compensatory-adaptive reaction of cells, develops in response to increased functional load due to hemodynamic disturbances in the lower extremity veins and to compensate for the quantitative deficit of SMCs resulting from their death. In “light” cells, ultrastructural alterations—such as the lysis of myofilaments, disorganization of organelles, dilated profiles of the granular sarcoplasmic reticulum, and solitary mitochondria with disorganized and lysed cristae—provide structural evidence of regenerative-plastic insufficiency and a low level of contractile capacity in SMCs under the conditions of a bioenegetic deficit. The ultrastructure of “dark” cells, featuring pronounced destructive changes in mitochondria and detachment of the basement membrane, may indicate damage to the SMC cytoskeleton. Collectively, such ultrastructural changes likely reflect irreversible degenerative alterations and signify cell death.

Qualitative analysis demonstrates that normal aging in elderly individuals features increasing connective tissue deposition between smooth muscle bundles. Varicose disease accelerates this process, with progressive collagen infiltration among muscle cells that intensifies with both disease duration and patient age. The smooth muscle cells themselves develop structural abnormalities including hypertrophy and phenotypic heterogeneity, accompanied by disrupted intercellular communication. Quantitative assessment reveals peak development of subintimal longitudinal smooth muscle cells during middle age—a pattern maintained in varicose patients. However, elderly and senile patients with varicose disease show significant reductions compared to both age-matched controls and younger patient groups, contributing to longitudinal venous wall stretching and tortuosity development.

While circular middle-layer smooth muscle cells maintain stable volumetric fractions during normal aging, varicose disease produces significant reductions, indicating impaired contractile function. The outer layer’s longitudinal muscle bundles normally increase with age, suggesting heightened functional importance. Young and middle-aged varicose patients exhibit elevated longitudinal muscle content versus controls, potentially representing compensatory adaptation. This protective mechanism fails in elderly varicose patients, who demonstrate marked reductions.

The obtained data enables a more in-depth investigation of the morphological and ultrastructural changes in the components of the venous wall, both in normal aging and in varicose disease. In addition to previously established knowledge, the findings of the present study allow for a comparative analysis of changes associated with natural aging and those associated with varicose disease. Nevertheless, the fundamental mechanisms underlying both vascular aging and varicose disease require further investigation.

The present study has several limitations. The research methodology does not allow for a correlation between the histological and ultrastructural data and in vivo ultrasound data, which would have increased the value of the obtained results. Furthermore, the biopsy collection procedure may somewhat alter the histoarchitectonics of the tissue, which must be noted.

## 5. Conclusions

Physiological aging of the great saphenous vein’s muscular components has several distinctive features: (1) development of subintimal longitudinal smooth muscle cells, peak in middle age (2) stable circular middle-layer muscle fractions, and (3) progressive increases in outer-layer longitudinal muscle bundles. Varicose disease accelerates sclerotic transformation across tissue, cellular, and subcellular levels, ultimately reducing all muscular components’ volumetric fractions through combined degenerative and fibrotic processes.

## Figures and Tables

**Table 1:** Distribution of the study material by age group, including sex composition, age ranges, and the number of morphological analyses performed on great saphenous vein fragments in both normal conditions and varicose disease cases.

Age Groups	Groups							
	Control				Varicose Disease			
	M	W	Age (Years)	n=	M	W	Age (Years)	n=
Young adults (18–44 years)	4	6	37 [35–38]	10	9	11	32 [26–34.5]	20
Middle-aged adults (45–59 years)	7	8	53 [49–57]	15	13	17	53.5 [50–55]	30
Old-aged adults (60–74 years)	8	7	70 [65–71]	15	10	10	68 [67–70]	20
Elderly (75–89 years)	7	8	82 [79–87]	15	4	6	79 [78–81]	10
Total	26	29		55	36	44		80

**Table 2:** Indicators of the volumetric fraction of transversely oriented smooth muscle cells of the middle layer of the great saphenous vein under age-related norms and in varicose disease.

Age Groups	Control	VD	*p* Value
1	82 [80–84]	77 [72–80]	*p* = 0.0038
2	80 [77–85]	71.5 [69–75]	*p* < 0.0001
3	79 [73–83]	34.5 [30–39.5]	*p* < 0.0001
4	80 [79–84]	29 [28–32]	*p* < 0.0001
P between age groups	*p* = 0.36	*p* < 0.0001; P1–2 *p* = 0.002; P1–3 *p* < 0.0001; P1–4 *p* < 0.0001; P2–3 *p* < 0.0001; P2–4 *p* < 0.0001; P3–4 *p* = 0.01	

**Table 3:** Indicators of the volumetric fraction of longitudinally oriented smooth muscle cells in the outer layer of the great saphenous vein under age-related norms and in varicose disease.

Age Groups	Control	VD	*p* Value
1	26.5 [26, 27]	40 [35.5–43]	*p* < 0.0001
2	34 [31–36]	42.5 [37–48]	*p* = 0.0014
3	40 [37–44]	39.5 [34.5–43]	*p* < 0.0001
4	41 [38–43]	28.5 [23–32]	*p* = 0.04
P between age groups	*p* < 0.0001; P1–2 *p* = 0.0001; P1–3 *p* = 0.0002; P1–4 *p* = 0.0001; P2–3 *p* = 0.00087; P2–4 *p* = 0.00019; P3–4 *p* = 0.64	*p* < 0.0001; P1–2 *p* = 0.078; P1–3 *p* = 0.89; P1–4 *p* = 0.0001; P2–3 *p* = 0.049; P2–4 *p* = 0.0001; P3–4 *p* = 0.0002	

## Data Availability

Data available on request from the authors.

## Abbreviations

The following abbreviations are used in this manuscript:

VD	varicose disease
CVI	chronic venous insufficiency
SMC	smooth muscle cell
GCV	great saphenous vein
